# Optimizing Surgical Management for Rhegmatogenous Retinal Detachment in Eyes with Active Retinoblastoma: A Safety-Driven Approach

**DOI:** 10.3390/jcm13092511

**Published:** 2024-04-25

**Authors:** Yacoub A. Yousef, Omar AlHabahbeh, Mona Mohammad, Hadeel Halalsheh, Mustafa Mehyar, Mario Damiano Toro, Ibrahim AlNawaiseh

**Affiliations:** 1 Department of Surgery (Ophthalmology), King Hussein Cancer Center, Amman 11941, Jordan; omarhabahbeh.oh@gmail.com (O.A.); dm.11804@khcc.jo (M.M.); mustafamehyar@hotmail.com (M.M.); 2 Department of Pediatric Oncology, King Hussein Cancer Center, Amman 11941, Jordan; hadeelhalalsheh@khcc.jo; 3 Chair and Department of General and Pediatric Ophthalmology, Medical University of Lublin, 20-079 Lublin, Poland; toro.mario@email.it; 4 Eye Clinic, Public Health Department, University of Naples Federico II, 80131 Naples, Italy

**Keywords:** retinoblastoma, retinal detachment, scleral buckle

## Abstract

**Introduction:** Intraocular surgeries are conventionally contraindicated for patients with active retinoblastoma (Rb) due to the potential risk of tumor dissemination. However, surgery is occasionally necessary to preserve vision in patients with a single eye when the eye is complicated by rhegmatogenous retinal detachment (RRD). **Objective:** This study aims to evaluate the outcomes of surgical repair for RRD in pediatric patients with active Rb utilizing a non-drainage scleral buckling approach. **Results:** This cohort included six eyes from six patients who harbored active Rb and presented with RRD; one had a concurrent tractional component. All eyes (100%) had active intraocular Rb and were undergoing active therapy (systemic chemotherapy, cryotherapy, and thermal laser therapy) when RRD developed. RRD consistently manifested at the site of recent cryotherapy in all cases. RRD repair in the affected eyes was performed by scleral buckling without subretinal fluid drainage. Five of the six eyes (83%) achieved complete retinal reattachment. One eye (17%) with a tractional component exhibited partial reattachment and was eventually enucleated due to persistent active disease. At a median follow-up of 15 months (range 12–180 months) after scleral buckling, all five eyes had persistent retinal attachment, and no case developed orbital or distant metastasis. **Conclusions:** Our study demonstrates that nondrainage scleral buckling is an effective and safe method for the surgical repair of RRD in eyes harboring active Rb, as most cases achieved persistent complete retinal reattachment without the risk of tumor spread.

## 1. Introduction

Retinoblastoma (Rb) is the most common primary intraocular malignancy in children, with an incidence of one in 15–20 thousand live births [1,2]. Multiple treatment modalities have been utilized for Rb, including chemotherapy (intra-arterial, systemic, and intravitreal), cryotherapy, laser therapy, plaque radiotherapy, and enucleation. Each modality has its indications, advantages, disadvantages, and associated risks [3,4,5,6,7,8,9,10]. Rhegmatogenous retinal detachment (RRD) is a reported rare complication that occurs when a retinal tear develops at the edge of a calcified tumor due to local treatments or necrosis of the retina after initiation of treatment [11,12]. Traditionally, these eyes have been enucleated when active disease is present. However, circumstances may arise where surgical repair of the retinal detachment becomes necessary, particularly in monocular patients for whom the contralateral eye is already enucleated. The optimal surgical repair approach and timing of repair for RRD remain a subject of debate, compounded by safety concerns to minimize the potential for viable tumor cell dissemination. Stevenson et al. reported three cases of local extraocular extension of Rb (following intraocular surgery via a transscleral approach) within a single center over 12 months [13].

There is no consensus on how to manage such a complication in Rb patients. Here, we describe our experience in managing RRD in eyes harboring active Rb using a minimally invasive approach that involves scleral buckling and intraoperative retinopexy without fluid drainage.

## 2. Methods

The Institutional Review Board approved the study, which is a retrospective case series of six eyes with clinical diagnoses of Intraocular Rb complicated with RRD. The study period spans January 2011 to December 2023 and involves patients treated at the King Hussein Cancer Center (Amman, Jordan) and Ibn Al-Haitham Hospital (Amman, Jordan). All cases were diagnosed by a vitreoretinal surgeon and ocular oncologist, and all surgeries were performed by the same vitreoretinal surgeon (IN).

Each patient underwent a comprehensive ophthalmic examination, performed under general anesthesia, which included various assessments, such as indirect ophthalmoscopy, ocular ultrasound B-scan, and brain magnetic resonance image (MRI) scan, as needed. Fundus photos were taken for documentation. Selection required access to patients’ medical and radiological reports and fundus RetCam images. The data collected included the patient’s age, gender, laterality, age at diagnosis, disease stage, treatment modalities, follow-up, outcomes, and best-corrected visual acuity (BCVA) at the initial and final examinations.

Regarding the inclusion and exclusion criteria, only the eyes presenting with RRD in conjunction with active intraocular Rb were included. Eyes with other types of retinal detachment (tractional or exudative) were excluded. Furthermore, patients who declined treatment or were monitored for less than six months were not included in the assessment of treatment outcomes.

### Surgical Technique

Informed consent was obtained from the parents of all patients pre-operatively, and all patients were operated under general anesthesia. One hour before the procedure, the patients received IV mannitol of a concentration of 20% at a dose of 5 mg/kg, given over 45 min. Then, after scraping and draping, a 360-degree conjunctival peritomy was performed. All four recti muscles were identified and hooked by placing a Gass retinal detachment hook and traction sutures (2-0 silk) beneath the insertions of the exposed recti muscles. After that, the 4 quadrants of the sclera were inspected using the indirect ophthalmoscope and a sterile 28 diopter biconvex indirect lens. Funduscopy was performed and all suspected areas of thin or frank retinal holes were treated with cryotherapy (Cryo Super Deluxe AA3 machine). Mild cryo reaction was applied until we reached a mild whitening of the retina; any suspected active Rb lesion was treated by the triple freeze-thaw technique. A localizing mark was made with a scleral marking pen on the point of the sclera overlying the posterior edge of the retinal break, and then a 240/2.5 mm silicon band was sutured to the sclera using a single matrix suturing technique with a 5-0 Ethilon suture at the posterior edge of the suspected holes and thin retina (previously marked). The silicone band was passed 360 degrees around the globe under the recti muscles. During all procedures, neither scleral external drainage nor anterior chamber paracentesis were performed (dropping of the IOP was managed using IV mannitol preoperatively, ocular massage intraoperatively, or acetazolamide tablets postoperatively). Postoperative drops included a tobramycin and dexamethasone mix four times daily and a mydriatic eye drop (cyclopentolate 1% or atropine 1%). Postoperative follow-ups involved clinical examinations under anesthesia to assess surgery success and retina attachment, in addition to ret-cam imaging for postoperative documentation, on the first day and then one week, one month, three months, and six months after surgery. Further follow-up visits depended on the condition of retinal detachment and Rb.

The same vitreoretinal specialist (IN) conducted all surgeries to ensure consistency, technique, and expertise.

## 3. Results

Our study included six eyes of six different Rb patients; none had a family history of Rb. These patients developed RRD during the treatment of active Rb. The median age at diagnosis was 25 months (range 12–48 months). Five of the six patients (83%) were male. Only one patient (17%) had unilateral Rb.

In this study involving six patients with Rb, five had bilateral disease, and one had unilateral disease. Out of the five patients with bilateral disease, three patients had a single eye (the other eye was previously enucleated for advanced intraocular Rb), and one more patient had to have her contralateral eye enucleated later on (after retinal detachment repair of the other eye) for recurrent massive disease. At the end of the study, out of six patients and six eyes treated for retinal detachment, three patients had a single eye and the retinal detachment was successfully repaired; one had bilateral disease and both eyes were salvaged; one had unilateral disease and the affected eye was salvaged; and unfortunately one, who presented with a single eye harboring retinal detachment on top of active Rb, was not successfully treated—the patient ended with bilateral enucleation. Overall, retinal reattachment was successfully achieved in five (83%) eyes, but unfortunately failed in one eye, which was eventually enucleated. The median follow-up time for all patients after the buckling procedure was 20 months (range 12–180 months). Table 1 provides a brief description of each case.

**Case #1:** A 2-year-old male was diagnosed with bilateral Rb. According to the International Classification of Retinoblastoma (ICRB), the right eye was group C, while the left eye was group E and was primarily enucleated. He received a total of six cycles of systemic chemotherapy (carboplatin, vincristine, etoposide (CVE)) coupled with focal treatment (laser and cryotherapy).

Four weeks after completing the final cryotherapy treatment, the patient experienced total RRD. A retinal break was identified at the margin of the calcified tumor. To address this, scleral buckling and intraoperative retinopexy with cryotherapy were performed without fluid drainage. Over the initial four weeks of follow-up, the retina reattached and tumor activity subsided after two additional sessions of thermal therapy (TTT). Subsequently, the retina remained attached throughout the next 15 years of follow-up, with no evidence of tumor recurrence or metastasis. The patient’s best corrected visual acuity at the last follow-up visit was 6/9.

**Case #2:** A 3-year-old boy was diagnosed with Rb in both eyes. Prior to being referred to our center, his right eye underwent enucleation, while his left eye received five cycles of systemic chemotherapy without focal therapy (three cycles of Carboplatin/Etoposide and two cycles of Cyclophosphamide/Vincristine). Upon arrival at our center, the left eye exhibited three active retinal tumors, each partially calcified with surrounding vitreous seeds. Treatment included transpupillary thermotherapy (TTT) laser, cryotherapy, and subtenon carboplatin injection.

**Figure 1:**
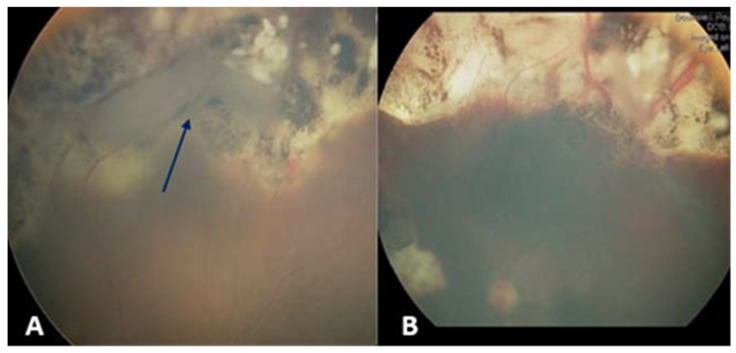
**(Case #2)** (**A**) Fundus photo showing the site of the retinal break at the margin of the calcified superior tumor (blue arrow). (**B**) Around three weeks post scleral buckle repair, the retina is reattached, and the remaining retinal tumors show a response to TTT laser sessions.

Four weeks after the fourth subtenon injection, he developed subtotal RRD, and a retinal break was identified at the margin of the calcified superior tumor (Figure 1A). Scleral buckling and retinopexy of the retinal break were implemented for retinal repair. The retina was reattached within three weeks, and the remaining retinal tumors were treated with four further TTT sessions (Figure 1B). Over an eight-year follow-up period post-surgery, there has been no evidence of tumor reactivation or metastases, and the patient’s best corrected visual acuity was 6/12 at the most recent examination.

**Figure 2:**
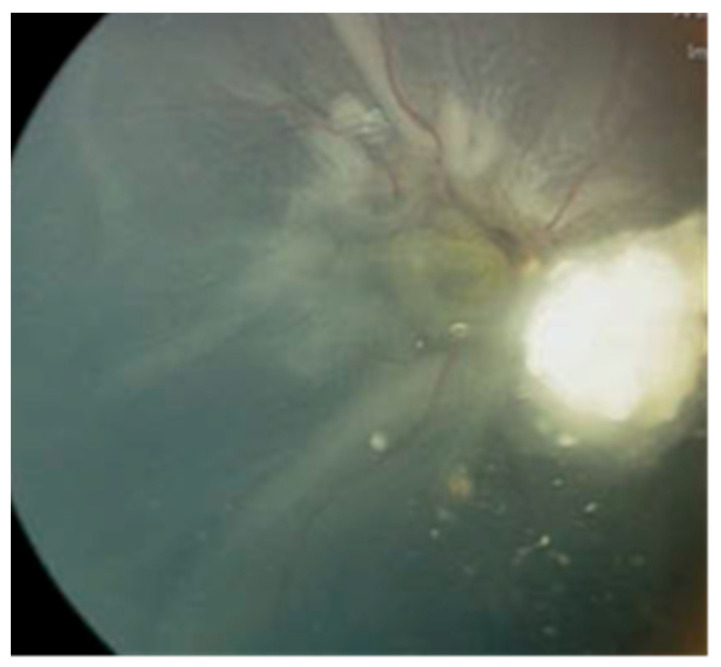
**(Case #3)** Fundus photo for the right eye shows active Rb with subtotal RRD and a tractional component.

**Case #3:** A 2-year-old male, diagnosed with bilateral Rb, had already undergone enucleation for the left eye in a different country. Before being referred to our center, he received six cycles of systemic chemotherapy to manage the active tumors in the right eye. Upon the first assessment at our center, the right eye exhibited active Rb, subtotal RRD, and a tractional component (Figure 2).

An effort was made to address the retinal detachment using a conventional approach involving scleral buckling and retinopexy without fluid drainage. While there was a minor improvement in the retinal detachment after the surgical procedure, the tumor displayed resistance to chemotherapy and continued to enlarge. The retina failed to reattach over a three-month postoperative follow-up period, and the tumor persisted in its growth. Due to the poor prognosis for vision and the heightened risk of metastasis, the decision was ultimately made to proceed with enucleation of the right eye.

**Figure 3:**
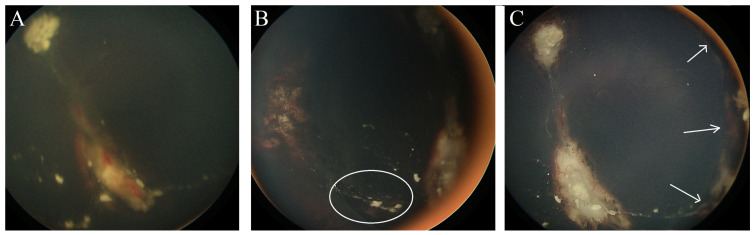
**(Case #4):** (**A**) Fundus photo of the left eye with active retinal tumor and RRD. (**B**) The site of the retinal break (white circle) is inferonasal. (**C**) Following retinal repair with scleral buckle, the retina is flat, and all tumors are inactive; white arrows show the edge of the scleral buckle.

**Case #4:** A 12-month-old male patient had bilateral Rb (right eye group D, left eye group C). He was given six cycles of systemic chemotherapy (CVE) coupled with focal consolidation therapy (laser and cryotherapy). In addition, he received intravitreal Melphalan injections for active vitreous seeds in the left eye. Three months after the initial presentation, he developed RRD in the left eye (Figure 3A), and a retinal break was found adjacent to the cryotherapy scar. Surgical retinal detachment repair was performed using a buckle without the need for fluid drainage, resulting in retina reattachment within four weeks (Figure 3B). After 18 months of follow-ups, the patient maintained a reattached retina with an inactive calcified tumor and inactive vitreous seeds (Figure 3C).

**Figure 4:**
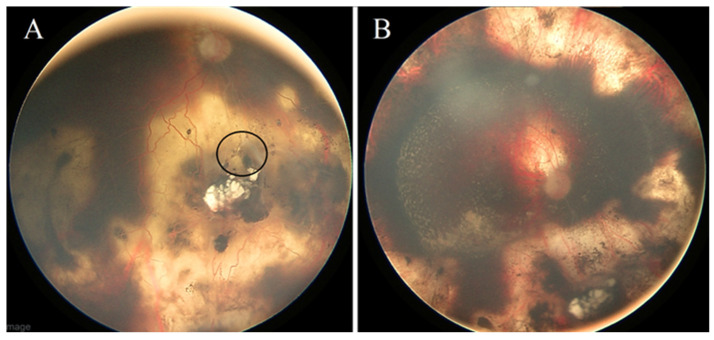
**(Case #5):** (**A**) The right eye had subtotal RRD due to a retinal break (black circle). (**B**) The retina is totally reattached after scleral buckle repair, and all tumors are dead.

**Case #5:** A 4-year-old male patient was diagnosed with unilateral Rb in the right eye (classified as ICRB group D). His treatment involved systemic chemotherapy (comprising eight cycles of CVE) combined with focal consolidation therapies (laser and cryotherapy). Additionally, the right eye received multiple intravitreal Melphalan injections to address active vitreous seeds. The tumor exhibited an excellent response to the aforementioned treatments. However, during a subsequent follow-up examination, the patient developed RRD (Figure 4A), which required scleral buckling with no need for fluid drainage. A follow-up eye exam 14 months later revealed that the retina remained attached with no indications of new tumor activity (Figure 4B).

**Figure 5:**
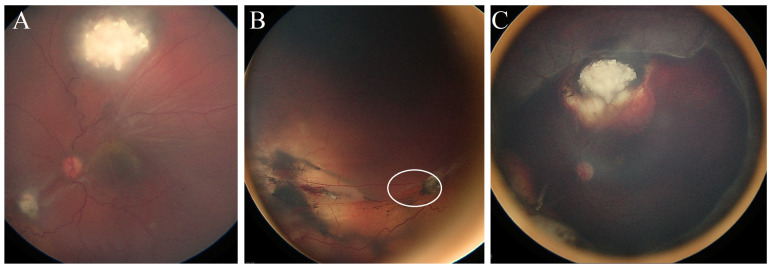
**(Case #6):** (**A**) Left eye fundus with active retinal tumors and retinal detachment involving the macula. (**B**) The circle indicates the inferonasal retinal break that confirmed the diagnosis of RRD. (**C**) In the same patient, the retina is reattached following repair with a scleral buckle, and all the tumors are inactive after focal therapy.

**Case #6:** A 16-month-old female patient was diagnosed with bilateral Rb (ICRB group D for both eyes). Initially, her treatment plan involved systemic chemotherapy and focal consolidation therapy. However, she later developed RRD in the left eye (Figure 5A), necessitating a scleral buckle procedure without fluid drainage. At her subsequent follow-up visit after three weeks, the left eye showed substantial retina reattachment. Throughout her follow-up appointments, she underwent multiple argon and TTT treatment sessions to address signs of tumor activity. Unfortunately, her right eye exhibited extensive recurrence involving retinal tumors and vitreous seeds. In response, intra-arterial Melphalan chemotherapy was initiated for her right eye, but regrettably, it did not yield a favorable response. After four weeks, the decision was made to proceed with enucleation of the right eye. At her most recent follow-up, conducted 15 months after the scleral buckle surgery, the retina remained flat and securely attached, with stable and inactive retinal tumors (Figure 5C).

## 4. Discussion

RRD has been reported as a possible complication in eyes with Rb due to retinal necrosis associated with systemic chemotherapy and local treatment [14]. Although it is a rare complication, it is seen more frequently with the combination of systemic chemotherapy, cryotherapy, and external beam radiotherapy [15]. It has also been reported as a rare complication after external beam radiation. It is thought that the development of prominent calcifications following treatment of the damaged and thinned retina may be responsible for the development of retinal breaks [12]. Conventionally, scleral buckling (SB), pars plana vitrectomy (PPV), and pneumatic retinopexy (PR) are the most commonly used techniques for the management of RRD [16]. However, given the unique nature of Rb and the associated risk of tumor dissemination, the available treatment modalities for RRD in eyes harboring active intraocular Rb are notably limited.

In our study, we included six eyes of six different patients who had RRD with active Rb. Three of these patients had a single eye at presentation, since the contralateral eyes were already enucleated due to Rb, accentuating the significance and value of retinal detachment management in these instances. The remaining cases included two patients with bilateral disease and one patient with unilateral Rb.

Our primary goals were to maintain the eye’s structural integrity and minimize the risk of disease dissemination. To achieve these goals, we refrained from puncturing the eye or draining subretinal fluid, as this would have allowed any remaining viable tumor cells to exit the eye, thereby raising the risk of metastasis and mortality [17]. We performed scleral buckling without drainage of subretinal fluid, and hence, we substituted any steps that required globe perforation, such as subretinal fluid drainage and anterior chamber paracentesis. For example, to reduce intraocular pressure (IOP) and prevent central retinal artery occlusion, we prescribed pre- and intra-op intravenous injections of Mannitol and post-op oral Acetazolamide. Out of six eyes, we successfully repaired the retinal detachment and maintained functional vision in five eyes (83%). The remaining eye, which did not respond favorably, had a tractional component that could not have been reattached because of mechanical traction, rather than the rhegmatogenous component.

The literature has described different techniques for managing RRD in Rb patients, including segmental buckling. These methods are associated with varying rates of anatomic reattachment and visual prognoses. Mullanoy and coauthors reported the case of a monocular 11-month-old boy who developed RRD following cryotherapy and external beam radiation, resulting from several areas of retinal breakdowns. The child underwent sclera buckling to reposition the detached retina without fluid drainage. However, AC paracentesis was performed to reduce IOP [11]. In a series of nine cases by Baumal et al., the RRDs were repaired either by taking an external approach with a scleral buckling procedure (with or without drainage of subretinal fluid) or by taking an internal approach with pars plana vitrectomy. Among these, seven eyes were repaired using a scleral buckling procedure. The subretinal fluid was drained externally in five eyes, whereas two eyes were repaired without subretinal fluid drainage; in one, the reattachment persisted, while in the other, it was redetached [18]. However, this publication did not address the risk of post-op metastasis after drainage of the subretinal fluid in this series.

Honavar et al. published a case series in which eight patients underwent scleral buckling procedures after completion of treatment for Rb: five without fluid drainage and three with fluid drainage. The surgery was successful in achieving retinal reattachment in six patients. However, one patient developed systemic metastasis. Honavar et al. also performed a standard 3-port pars plana vitrectomy for 12 eyes in this series. The indications for vitrectomy included vitreous hemorrhage in eight eyes and RRD with proliferative vitreoretinopathy in two eyes. Among the 12 eyes, five experienced Rb recurrence, eight necessitated enucleation, and two had systemic metastasis afterward. This led the authors to conclude that vitrectomy may be associated with a higher risk of recurrence, enucleation, and systemic metastasis [19]. In support of this conclusion, Lu Li and coauthors studied the results of pars plana vitrectomy in treating intraocular Rb in 28 children. One patient died due to tumor recurrence and brain metastasis, and another 10 eyes were ultimately enucleated because of uncontrolled tumors and the potential for distant metastasis [20]. Moreover, Shen and coauthors documented three cases of initially misdiagnosed Rb who underwent PPV and evisceration as cases of coats disease, blunt trauma, and endophthalmitis. The diagnosis of Rb had been confirmed later, and two of the cases received adjuvant orbital radiotherapy. However, all three cases died of systemic tumor metastases [21]. Due to this fatal risk of tumor dissemination, our approach aimed to avoid any form of globe penetration or fluid drainage. Likewise, Buerk et al. documented two cases of Rb in children who had experienced RRD after receiving chemotherapy and cryotherapy treatment. In both instances, the affected eyes underwent scleral buckling surgery without draining subretinal fluid, and intraoperative retinopexy of the retinal break was not performed. Following surgery, the retinas were successfully reattached. During subsequent examinations under anesthesia, retinopexy using indirect laser photocoagulation was conducted. The retinas remained attached throughout the follow-up period, ranging from 2 to 5 years [22]. In a retrospective case series reported by Saumya and colleagues, nine eyes of nine patients were diagnosed with RRD. Seven of these eyes underwent a non-drainage scleral buckling procedure. On the other hand, three eyes underwent vitreous surgery; in two of these eyes, tumor excision was performed alongside retinectomy using a Melphalan infusion, and a silicone oil tamponade was eventually employed. Retinal reattachment was achieved in all eyes, with eventual tumor control in seven eyes [23].

Shields et al. published a report for 10 patients with RRD following Rb treatment. The surgical procedures performed to repair RRD were recorded, including laser barricade, scleral buckle procedure with or without drainage, and pars plana vitrectomy (PPV). Only one eye initially underwent treatment with a scleral buckle without drainage. However, it continued to exhibit persistent detachment, necessitating a secondary PPV procedure with a silicone oil tamponade and resulting in persistent total retinal detachment [3]. Moreover, in a series of cases by Elaraoud, four patients developed RRD within a relatively short timeframe, approximately four to six weeks of focal therapy (cryotherapy or laser thermotherapy) for the Rb. The management approach involved an external procedure featuring a scleral buckling procedure and cryotherapy to repair the RRD. Additionally, an anterior chamber paracentesis was performed to ensure that the optic nerve head was perfused. The retina was reattached within eight weeks in three of the four operated patients. In the fourth patient, the retina did not reattach despite the intervention [24].

Although our study provides valuable insights for the ophthalmic oncology community into managing this rare complication within a rare disease without exacerbating the risk of metastasis, it is important to acknowledge several limitations. These include the study’s retrospective nature, its small patient cohort, the absence of clear guidelines regarding the timing of intervention, and the lack of a comparative group. Therefore, there is a pressing need for prospective, well-controlled, and more extensive multicenter studies to comprehensively assess both the efficacy and safety of our surgical treatment approach for repairing retinal detachment in eyes affected by active Rb.

## 5. Conclusions

RRD is a known complication that can develop during the course of Rb treatment, presenting a challenging scenario for ophthalmic surgeons. It is imperative to approach RRD in eyes with Rb with caution, as the goal is not only to repair the detachment, but also to preserve the eye and minimize the risk of metastasis and mortality associated with this malignancy. In managing RRD in Rb eyes, employing meticulous surgical techniques becomes paramount. One such approach involves scleral buckling without subretinal fluid drainage, which has been proven beneficial and safe in some instances. This technique offers advantages over traditional methods, which involve draining subretinal fluid externally. Avoiding direct manipulation of the subretinal space minimizes the risk of disseminating live tumor cells, which is crucial for preventing tumor dissemination and subsequent metastasis.

Moreover, it reduces the likelihood of intraocular hemorrhage and the potential for incarceration of retinal or vitreous tissue, thereby preserving the structural integrity of the eye. Furthermore, the technique of scleral buckling without subretinal fluid drainage has the added benefit of not exacerbating proliferative vitreoretinopathy in the event of surgical failure. This is particularly advantageous in cases in which the underlying Rb pathology may predispose the eye to such complications. However, it is essential to acknowledge that this approach may not be universally applicable, especially in cases where mechanical traction contributes to retinal detachment, as observed in certain instances.

## Figures and Tables

**Table 1:** Summary of tumor features and treatment outcomes for six eyes with active intraocular retinoblastoma that had RRD ^#^ and were treated by scleral buckle without subretinal fluid drainage.

	Gender	Age at Diagnosis (Months)	Laterality	Status of the Other Eye	ICRB *	Previous Treatments	Result of RRD Repair	Eye Salvage	Visual Outcome	Follow Up Duration (Months)
1	Male	24	Bilateral	Enucleated	C	Systemic chemotherapy, TTT ^@^, Cryotherapy	Attached	Salvaged	6/9	180
2	Male	36	Bilateral	Enucleated	C	Systemic chemotherapy, TTT, Cryotherapy, subtenon Carboplatin	Attached	Salvaged	6/12	96
3	Male	22	Bilateral	Enucleated	C	Systemic chemotherapy	Failed	Enucleated	-	12
4	Male	12	Bilateral	Group D	C	Systemic chemotherapy, Laser, Cryotherapy, Intravitreal Melphalan.	Attached	Salvaged	Follows objects	18
5	Male	48	Unilateral	Normal	C	Systemic chemotherapy, Laser, Cryotherapy, intravitreal Melphalan.	Attached	Salvaged	6/60	14
6	Female	16	Bilateral	Enucleated	D	Systemic chemotherapy, TTT, cryotherapy.	Attached	Salvaged	Follows objects	15

* ICRB: International Intraocular Retinoblastoma Staging System. ^#^ RRD: Rhegmatogenous Retinal Detachment. ^@^ TTT: Transpupillary Thermal Therapy.

## Data Availability

Data are available on reasonable demand to the corresponding authors.
